# Orientation of Airborne Laser Scanning Point Clouds with Multi-View, Multi-Scale Image Blocks

**DOI:** 10.3390/s90806008

**Published:** 2009-07-29

**Authors:** Petri Rönnholm, Hannu Hyyppä, Juha Hyyppä, Henrik Haggrén

**Affiliations:** 1 Institute of Photogrammetry and Remote Sensing, Helsinki University of Technology, P.O. Box 1200, FI-02015 TKK, Finland; E-Mails: hannu.hyyppa@tkk.fi; henrik.haggren@tkk.fi; 2 Department of Remote Sensing and Photogrammetry, Finnish Geodetic Institute, P.O. Box 15, 02431 Masala, Finland; E-Mail: juha.hyyppa@fgi.fi

**Keywords:** laser scanning, photogrammetry, registration, ICP method, interactive orientation, multi-view, multi-scale

## Abstract

Comprehensive 3D modeling of our environment requires integration of terrestrial and airborne data, which is collected, preferably, using laser scanning and photogrammetric methods. However, integration of these multi-source data requires accurate relative orientations. In this article, two methods for solving relative orientation problems are presented. The first method includes registration by minimizing the distances between of an airborne laser point cloud and a 3D model. The 3D model was derived from photogrammetric measurements and terrestrial laser scanning points. The first method was used as a reference and for validation. Having completed registration in the object space, the relative orientation between images and laser point cloud is known. The second method utilizes an interactive orientation method between a multi-scale image block and a laser point cloud. The multi-scale image block includes both aerial and terrestrial images. Experiments with the multi-scale image block revealed that the accuracy of a relative orientation increased when more images were included in the block. The orientations of the first and second methods were compared. The comparison showed that correct rotations were the most difficult to detect accurately by using the interactive method. Because the interactive method forces laser scanning data to fit with the images, inaccurate rotations cause corresponding shifts to image positions. However, in a test case, in which the orientation differences included only shifts, the interactive method could solve the relative orientation of an aerial image and airborne laser scanning data repeatedly within a couple of centimeters.

## 1. Introduction

Accurately measured 3D information from our environment has become more and more important in our daily life. For example, virtual environments, environmental and urban planning, decision making processes, and modern navigation systems require 3D data that correspond with reality. Laser scanning has become popular due to its fast 3D point cloud acquisition, improvements in post processing software and high usability of the data generated. Photogrammetric techniques have been used for a long time, but development of digital cameras, more efficient automatic and semiautomatic 3D measuring methods and good internal geometry have been key factors that have kept image-based techniques as suitable alternatives for 3D modeling. Both methods have advantages and disadvantages, and in many ways they complement each other.

Complete 3D modeling requires both terrestrial and airborne data [1]. In addition, both perspectives should preferably include both laser scanning data and images. The viewing direction of a data acquisition method defines which parts of a building can be modeled reliably. A terrestrial point of view does not allow seeing all roof shapes, and nadir perspective prevents accurate modeling of vertical structures. Thus, different perspectives offer additional information about the behavior and quality of data. Therefore, terrestrial images are also excellent for assuring the quality of airborne laser scanning data [2–5].

Airborne laser scanning data is widely used for, e.g., creation of digital terrain models (DTM) (e.g., [6–11]) and for extracting buildings (e.g., [12–15]), other infrastructure such as bridges (e.g., [16]), as well as urban trees (e.g., [17]). However, the point density of airborne laser scanning is typically not enough for the most accurate modeling, such as exact positioning of building outlines [18]. Terrestrial laser scanning provides significantly denser point clouds and good accuracy [19], but suffers from stationary data acquisition that makes the method slow and costly when large areas are modeled. Vehicle-based mobile laser scanning provides faster data acquisition and relatively dense point clouds, but suffers from satellite visibility problems in urban areas resulting in systematic errors of the order of 0.1–3 meters [20,21], in the data.

Integration of data obtained by different measuring methods enables numerous applications [4], but requires accurate relative orientations. The more detailed the 3D model needed, the more accurate the relative orientation required. Direct sensor orientation with GPS and inertial equipment is essential for laser scanning data acquisition and can also be used for detecting exterior orientations of images. Accuracies as high as 5–10 cm in position and better than 0.006° for ω and φ, and 0.01° for κ in rotations [22–25] have been reported. However, studies with photogrammetric frame sensors [24,26,27] have shown that insufficient satellite visibility, an incomplete relative orientation between the imaging sensor and GPS/IMU-components, inaccuracies of an imaging model, and transformations between various coordinate systems can reduce this accuracy. In general, direct sensor orientation alone is not yet providing orientations fine enough for most accurate integration of data from multiple sources [23].

Images are typically oriented in large image blocks by block adjustment. The main alternative for solving exterior orientations of aerial images is aerial triangulation utilizing image points, 3D ground features and possibly direct georeferencing observations of orientations [28]. The aerial triangulation requires manual identification of ground control points from the images, but the advantage of the method is that inaccuracies of interior orientations are compensated with self-calibration during the adjustment [29]. Multi-view image blocks, which consist of aerial and terrestrial images, are usually utilized when the size of an object to be modeled, such as a historical building, is convenient. Especially, the development of unmanned aerial vehicle (UAV) systems has recently increased the use of such multi-view image blocks (e.g., [30]). If images within a block differ much in scale, the resolution of images may reduce the interpretation accuracy, which should be taken into account. The use of multi-scale image blocks is discussed more in detail, e.g., in [31].

If the internal geometry of a terrestrial laser scanner has been calibrated and corrected [32], 3D laser scanning point clouds from multiple scans can be registered, e.g., using the iterative closest point (ICP) method or by extracting and matching tie features. The term registration means finding the geometric transformation which makes corresponding locations in the two 3D data sets [33]. ICP methods are very popular for registering two 3D data sets at the object space. An ICP algorithm minimizes point-to-point [34] or point-to-surface distances [35]. The method has several variants, which have been discussed, e.g., in [36,37]. Alternatively, several methods to register 2.5D and 3D surfaces have been reported [37]. Currently, many laser scanning software packages include ICP-based registration algorithms. Registered laser point clouds can reliably be transformed into a ground coordinate system using signalized ground control points.

Airborne laser scanning missions usually include several laser scanning strips. By applying a strip adjustment, the internal quality of laser scanning data can be improved [38]. This step is necessary in order to ensure homogeneous quality within all laser scanning strips. When airborne laser scanning data is transformed into a ground coordinate system, ground control points should be signalized with large targets since the point density is typically much lower than with terrestrial laser scanning. Large circular targets have been suggested for ground control points, e.g., in [39] and [40], but the horizontal accuracy depends highly on point density [39]. However, the correct height for the laser scanning point cloud is much easier to solve than horizontal shifts [10]. Alternatively to circular targets, pavement markings have been used as control points (e.g., [41,42]). The use of linear features has been studied, e.g., in [13,43,44], in which breaklines were extracted by finding intersections of two planes that were fitted to laser scanning data. Corresponding breaklines were extracted from images for orientation. In [45], a triangulation of multi-sensor data using straight lines and planar patches as tie features was presented. Also, the centroids of rectangular roofs have been suggested as tie features for the orientation of multi-sensor data [46]. The concept of registering airborne laser scanning data and images through photogrammetrically-derived 3D surfaces measured from stereo images has been reported by, e.g., [47,48].

One difficulty with multi-sensor orientation is that it can be a challenging task to find accurate corresponding features, if, e.g., resolution, perspective or the nature of data sets differs much. As an alternative to numerical methods, paper [2] describes how a single terrestrial image and airborne laser scanning data can be relatively oriented using an interactive orientation method. During interactive orientation, an operator adjusts a laser point cloud visually with the images by changing image orientation parameters and using anchor points. This being a manual method, an operator is able to interpret laser hits coming from both large objects and small objects. In many numeric methods, small details are usually considered as outliers and therefore filtered out even if they were to include significant information for orientation. Large objects, however, are especially suitable when laser scanning data is not dense, because shapes of point clouds can be used as interpretable tie features. In this article, an interactive orientation method is developed to use an image block instead of a single image. The advantage of using an image block is that a multiple viewing geometry gives more information about the orientation than a single viewing direction.

The objectives of the article are: 1) to demonstrate a method for an interactive orientation of multi-source and multi-scale data, 2) to compare orientation results of the depicted interactive method with reference image orientations, 3) to get a laser point cloud and a reference image block into the same coordinate system using the ICP method and 4) to integrate multi-source data.

## 2. Materials

**Figure 1.**
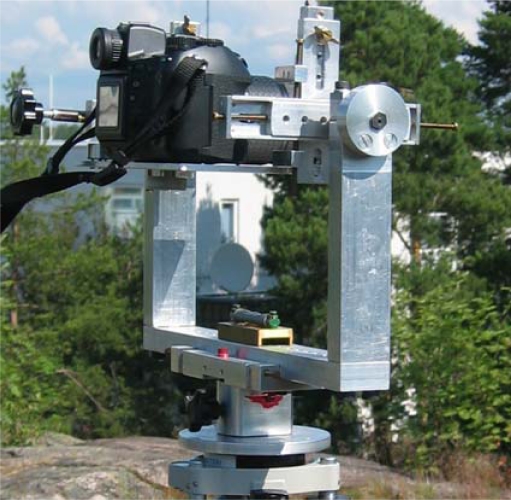
In order to create a measureable panoramic image, a concentric image acquisition was ensured with a calibrated camera mount.

The test area was located on the campus area of the Helsinki University of Technology (TKK) in Otaniemi. Test data included terrestrial images, a panoramic image, an aerial image, airborne laser scanning data, terrestrial laser scanning data, and total station measurements. Terrestrial images were taken with Olympus E-10 and Nikon D200 with image sizes of 2,240 × 1,680 and 3,872 × 2,592 pixels, respectively. A panoramic image was created from a set of concentric images taken with Olympus Camedia C-1400 L. A total of 7 images were stitched together into a rectilinear projection resulting in an image size of 10,729 × 5,558 pixels. In order to ensure concentric image acquisition, a special panoramic mount [49] was used (Figure 1). The number of other terrestrial images, acquired with Nikon D200, was 35.

A low-altitude aerial image was taken from the altitude of 200 m with a Hasselblad Landscape camera. The size of the sensor was 3,056 × 2,032 pixels and therefore the footprint of a single pixel on the ground was 4–4.5 cm, depending on the height of the object. The interior orientations of all cameras were known.

Airborne laser scanning data was acquired with a TopEye MK I helicopter-borne laser scanner. The flying altitude was 200 m, resulting in the average point density of 2–3 points/m^2^. The scan angle of the TopEye MK I laser scanner was ±20°, the wavelength 1.064 μm and the pulse repetition rate 7 kHz. From several laser scanning strips, only two parallel, partially overlapping strips were used. The strip adjustment was calculated using TerraMatch software yielding to a total RMS (dz) of 2.4 cm.

Terrestrial laser scanning data was acquired with a Faro LS 880 HE80 instrument. The scanner is able to achieve a measurement rate of 120,000 pulses/s, at maximum. In our experiment, the ¼ resolution of the maximum scanning resolution was used. Due to the 360° horizontal and 320° vertical coverage per scan position, the scanner is able to scan almost a complete hemisphere. The wavelength is 785 nm, and the linearity error has been reported to be 3 mm at the distance of 25 m and with a target having 84 % reflectivity (see www.faro.com). The location of the Faro scanner was selected in a way that its data completed those vertical surfaces, which had only very few hits from the airborne laser scanning.

In order to obtain all data sets into the local coordinate system, a total of 44 targets were measured using a Leica TCA 2003 total station. Leica’s 2 × 2 cm targets were modified to make them suitable also for photogrammetric measurements by framing the targets with black self-adhesive sticker paper. In addition, 21 non-reflective photogrammetric targets were placed on the scene and used as tie points in order to assist with the orientation of the photogrammetric image block.

## 3. Methods

### 3.1. Workflow

The reference orientations of images were solved in a bundle block adjustment of a multi-view, multi-scale image block. A relative orientation between the reference image block and airborne laser scanning data was calculated using the ICP method between a photogrammetrically-derived 3D model and a laser scanning point cloud. After the ICP registration, laser scanning data and the reference image block were in the same coordinate system. The result of the relative orientation was verified visually by superimposing laser scanning data onto aerial, terrestrial close-range and panoramic images.

**Figure 2.**
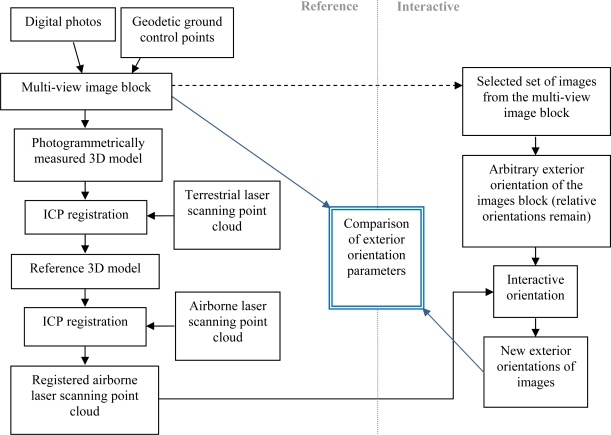
The workflow of the research.

In order to test the interactive orientation, an aerial image, a panoramic image and a close-range image were selected from the reference image block. Interior orientations were known from camera calibrations and relative orientations of all images from a bundle block adjustment. An initial exterior orientation of selected block of images was randomly chosen in such a way that the relative orientations of images were not changed. The selected images were oriented with the airborne laser scanning point cloud using the interactive method. Because the airborne laser scanning data was in the same coordinate system as the reference image block, the resulting orientations of the interactive method were comparable with the reference orientations of the original reference image block. The workflow is illustrated in Figure 2.

### 3.2. Reference Orientation for Laser Scanning Data

**Figure 3.**
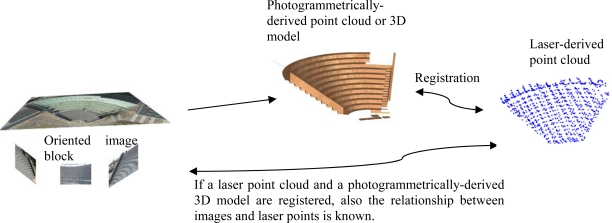
The relative orientation of an image block and a laser point cloud can be solved through the 3D object space.

In order to obtain a reference orientation for laser scanning data, an airborne laser scanning point cloud was registered with a photogrammetrically-derived 3D model, which was selected to be the reference surface. The photogrammetric 3D model was measured using a multi-scale image block, which included both aerial and terrestrial images. The orientation of the photogrammetric 3D model was known by the ground control points. Figure 3 illustrates how a relative orientation between images and a laser scanning point cloud is known if an image-derived 3D model is registered with a laser point cloud at the object space.

In our experiments, the ICP algorithm in Geomagic Studio software was used. This algorithm minimizes the distance between a point cloud and a surface. The reference surface was created mainly from photogrammetrically-derived point clouds. However, in order to add more features having different orientations, also terrestrial laser scanning data was used. Before extracting surfaces from the terrestrial laser scanning data, it was registered with the photogrammetric reference surface using the ICP method. This task was significantly easier than registering airborne laser scanning data with the photogrammetric 3D model. The main reason for that was the high point density of the terrestrial laser scanning data.

In practice, any 3D data sets that are used for the ICP registration should correspond to each other as well as possible, because the method can be sensitive to outliers [50]. In addition, a good initial registration is needed in order to prevent iteration to stop at a local minimum. Registration areas should contain enough distinguishable features having slopes to several directions in order to assure correct registration. Typically, outliers should be filtered out and only most reliable data sets should be used.

Orientations of images were solved in a bundle-block adjustment of 37 images using 848 natural tie points and 35 signalized points. The multi-scale image block consisted of close-range images, a panoramic image and a low-altitude aerial image. The reference orientation of the image block was solved using the iWitness software [51] resulting in the overall accuracy of 1.3 cm. The estimated accuracy of image referencing was 0.72 pixels. iWitness recalculates a complete block adjustment each time when a new observation is added. Therefore, the accuracy of the previously calculated 3D model may decrease, if more inaccurate images, such as aerial images, are included in the adjustment. To achieve as accurate 3D model as possible, the first image block included only terrestrial close-range images. 3D model points measured from close-range images were re-imported in the software as ground control points, before the aerial image and the panoramic image were included. For the ICP registration, only such tie points were selected that belong to enclosed features and all unconnected points were manually discarded. Unconnected points were, however, used as tie points when the aerial image was oriented into the image block.

**Figure 4.**
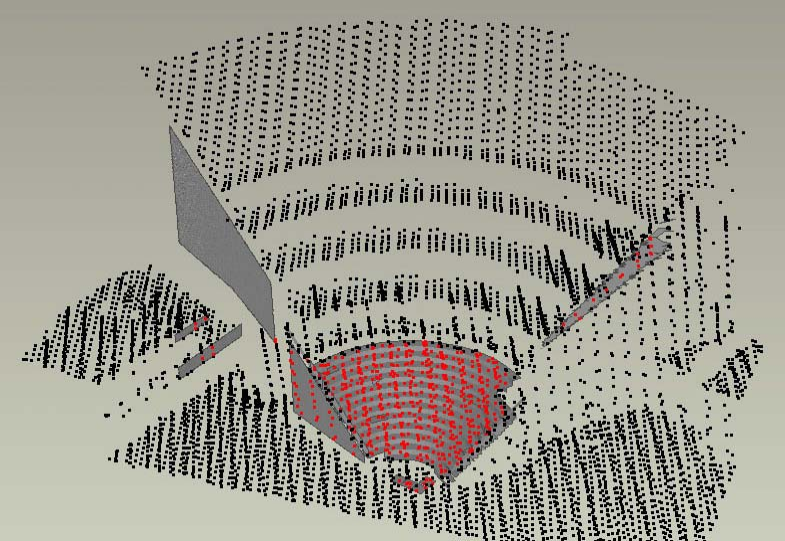
ICP registration results. The reference surface was a combination of photogrammetric and terrestrial laser scanning data. Only red laser points were included in registration.

As Figure 4 illustrates, only selected points were included in the ICP registration. After the registration, the software reported an average deviation of 2.5 cm. The relative orientation was examined by superimposing registered laser scanning data onto close-range images. Visual inspections did not reveal any significant errors in the relative orientation.

### 3.3. The Interactive Orientation of a Laser Point Cloud and a Multi-view Image Block

**Figure 5.**
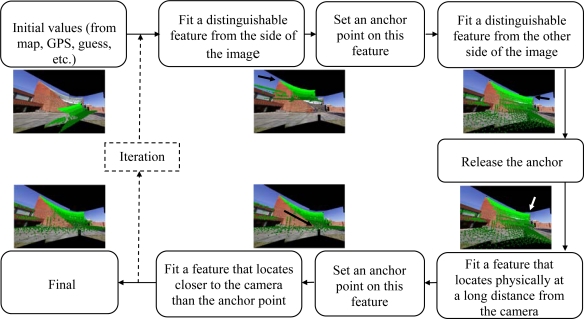
A suggestive workflow for an interactive orientation of a single image.

In this research, the interactive orientation method [2] was extended to be able to handle more than one image during the orientation. The interactive orientation method includes tools for changing exterior orientation parameters as well as for setting and using anchor points. For orientations, a complete laser point cloud or a selected subset of laser points can be used as a tie feature. The usability of the method is at its best with airborne laser scanning data, when a coarse sub-sampling of the scene usually makes it difficult to extract accurate tie features. Figure 5 illustrates a typical orientation workflow in the case of a single panoramic image.

Interactive orientation of a single image can be extended to consist of an image block. In that case, if orientation parameters of any individual image are changed, all orientations of other images from the image block are calculated and updated. When the location of an active camera is shifted, all other cameras are moving along with same amount of shift. The case of rotations is more complex, because a rotation of an active image causes both shifts and rotations to all other images. Because 3D rotation matrices describe a relationship between a camera and the ground coordinate system, we have to calculate the changed 3D rotation matrices through the ground coordinate system.

**Figure 6.**
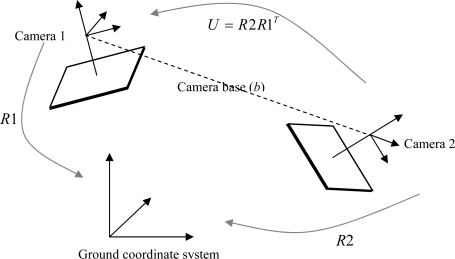
With 3D rotation matrices R1, R2 and U, camera coordinate observations can be rotated to a coordinate system parallel to the target coordinate system. Because 3D rotation matrices are orthogonal, inverse matrices can be calculated with matrix transposes.

For clarity, we present here a case of two images, although the calculation of all other images of the block is similar. In this example, Camera 1 is an active image, whose orientation parameters are changed interactively and the orientation of Camera 2 is calculated. At first, we define that 3D rotation matrices *R*1 and *R*2 realize transformations from the cameras to the ground coordinate system (Figure 6).

In addition, 3D rotation matrix *U* is the relative rotation between the two camera coordinate systems. Therefore, the equation describing a rotation between the camera coordinate systems is:

(1) $$U_{\mathrm{relative}}={R2}_{\mathrm{original}}\;{R1}_{\mathrm{original}}^{T}$$

If camera base (*b*) at the ground coordinate system is the difference between projection centers *P*1_0_ and *P*2_0_:

(2) $$b_{\mathrm{ground}}={P2}_{0\_\mathrm{original}}-{P1}_{0\_\mathrm{original}}$$

then the camera base converted from the ground coordinate system into the camera coordinate system of Camera 1 is:

(3) $$b_{\mathrm{camera}1}={R1}_{\mathrm{original}}^{T}b_{\mathrm{ground}}$$

Relative 3D rotation matrix *U_relative_* and camera base *b_camera_*_1_ remain the same no matter how an active image is rotated or shifted. If we rotate the active camera, we get a new 3D rotation matrix, *R*1*_new_*. The coordinates of the projection center of Camera 2 at the ground coordinate system can be calculated from:

(4) $${P2}_{0\_\mathrm{new}}={R1}_{\mathrm{new}}\;b_{\mathrm{camera}1}+{P1}_{0\_\mathrm{original}}$$

Using the new 3D rotation matrix of Camera 1 and the original relative 3D rotation matrix *U*, we can solve a new 3D rotation matrix for Camera 2:

(5) $${R2}_{\mathrm{new}}=U_{\mathrm{relative}}\;{R1}_{\mathrm{original}}$$

As a special case, if images fulfill the conditions of a normal case of stereo photogrammetry, 3D rotation matrices of Cameras 1 and 2 are identical and only a new location for the projection center of Camera 2 needs to be calculated (Equation 4).

The strategy for the interactive orientation of the image block is different, depending on whether there is a need to solve only shifts or both rotations and shifts. In the case that orientation differences include only shifts, all images may be employed as a master image by turns. The strategy can be that the first master image is registered along the x and y axes of the image coordinate system as well as possible. Then another image is selected as a master image and an orientation similar to that in the case of the first master image is completed. Any image can be the master image and the selection of the master image depends on optimum visibility. Finally, the orientation iterates to the final solution when orientation cannot be improved any more from any image.

Most commonly, both rotations and shifts need to be solved. If initial orientation is not close to the correct one, interactive orientation begins with the strategy presented in Figure 5. When the orientation is well enough and laser data is visible in all images, it is recommended to use one image as the master image and to use the other images only for monitoring. After changing the orientation parameters of the master image, superimposing laser scanning data onto the other images reveals whether changes had a positive influence on the relative orientation. In our experience, use of an anchor point at a well detectable feature usually assists with the orientation. If the anchor point is set, shifts along the X and Y axes of the camera coordinate system automatically change rotations. Again, by monitoring other images the correct directions of corrections can be detected. The optimal case would be when the viewing directions of images are perpendicular to each other. However, it is not usually possible to arrange such image acquisition. If the viewing directions are not perpendicular to each other, the shifts and rotations of the master image cause changes to all rotations and shifts along axes of the camera coordinate systems of other images. In other words, it is not always easy to predict how orientation changes of a master image affect the orientations of other images. Therefore, it is recommended not to try and fit features exactly to correct locations during correction of one rotation or shift direction, but only move them closer towards a better solution. By iterating, the solution becomes closer and closer to the correct solution until superimposing laser data onto images reveals no more orientation differences between the images and laser scanning data.

### 3.4. Applying Transformations to Laser Point Clouds

To transfer photogrammetric data accurately to the ground coordinate system is easier, in many cases, than transferring laser scanning data. For photogrammetric data there exist standard methods, such as bundle block adjustment, to find correct orientations. The interactive orientation method, however, is more flexible if the image orientation parameters are manipulated instead of the laser point cloud orientation. After interactive orientation, inverse transformation can be applied, in which the camera orientation is fixed to be the original one and the laser scanning point cloud is transformed according to the results of interactive orientation. First, laser scanning points (*x =* [*X Y Z*]*^T^*) are shifted in such a way that the origin of the coordinate system is at the projection center of a camera after an interactive orientation (*P*_0_*after*_):

(6) $$x_{\mathrm{shifted}}=x_{\mathrm{original}}-P_{0\_\mathrm{after}}$$

Next, the laser point cloud is rotated around the projection center using the relative 3D rotation matrix *U* (Equation 1), in which *R1* is a 3D rotation matrix after interactive orientation and *R2* is the original 3D rotation matrix. At the same time, possible shifts:

(7) $$t={\left[\begin{array}{lll} X_{0} & Y_{0} & Z_{0} \end{array}\right]}_{\mathrm{original}}^{T}-{\left[\begin{array}{lll} X_{0} & Y_{0} & Z_{0} \end{array}\right]}_{\mathrm{after}}^{T}={\left[\begin{array}{lll} {\mathrm{dX}}_{0} & {\mathrm{dY}}_{0} & {\mathrm{dZ}}_{0} \end{array}\right]}^{T}$$

between locations of projection centers before and after orientations can be corrected:

(8) $$x_{\mathrm{transformed}}={\mathrm{Ux}}_{\mathrm{shifted}}+t$$

Finally, the laser point cloud is shifted to the ground coordinate system:

(9) $$x_{\mathrm{final}}=x_{\mathrm{transformed}}+P_{0\_\mathrm{after}}$$

As a result, the image orientation is the original one and the laser point cloud is transformed into the same coordinate system according to the results of the interactive orientation between the image and the laser point cloud. An example of inverse transformation is presented in Section 4.3.

## 4. Results and Discussion

### 4.1. The Accuracy of an Interactive Orientation of Multi-view Image Blocks and Laser Scanning Data

Accuracies were examined by comparing interactive orientations with reference orientations. At first, a panoramic image and an aerial image were selected from a larger image block. From the block adjustment, the relative orientations of images were known. An interactive orientation of the image block and laser scanning data was completed eight times, starting each time from an arbitrary chosen initial orientation. The results from the interactive orientation were compared with the reference orientations (Table 1). The maximum shift of eight individual orientations was 30.5 cm indicating that the orientation had not been perfect in all cases. One reason was that the number of features and density of laser scanning data had not enabled accurate detection of rotations. If the rotations are not solved correctly, errors are also visible at camera locations. The location of the panoramic image was detected more accurately than the location of the aerial image because of the proximity of the former to the laser point cloud and its more illustrative viewing perspective. In the case of aerial image, the interpretation of the airborne laser scanning point cloud is not as clear as from the side view because of the acquisition perspective. Both interpretation difficulties and the resolution of the aerial image reduce the final orientation accuracy.

The second experiment included a panoramic image, an aerial image and a terrestrial close-range image. In this case, the close-range image was taken in such a way that the viewing direction was almost perpendicular to the viewing direction of the panoramic image.

**Figure 7.**
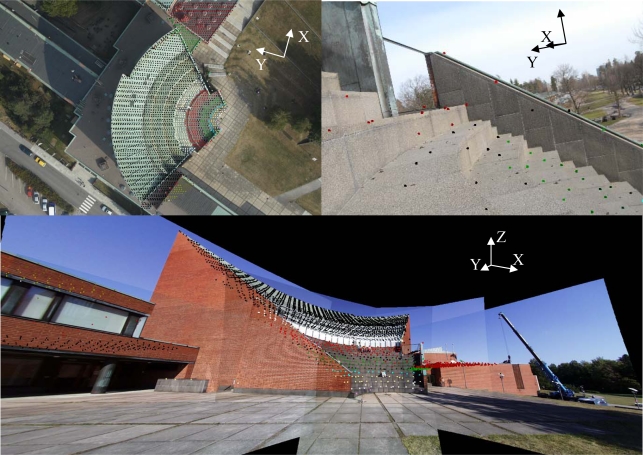
Laser scanning data, which was used for interactive orientation, superimposed onto aerial, close-range and panoramic images. The color-coding is illustrating the heights of laser points. The coordinate axes illustrate the approximate directions of the ground coordinate system.

The previous example revealed that the panoramic image had more shift along the viewing direction (close to the direction of the Y axis, see Figure 7) than in the other directions. It was predicted that the close-range image with the perpendicular viewing direction to the viewing direction of the panoramic image could reduce uncertainty in this particular direction. As the results illustrate (Table 2), practical examples were in conjugation with this prediction.

The location of the panoramic image has a maximum shift of 1.3 cm at the direction of the Y axis of the ground coordinate system when compared with the reference orientation. A disadvantage of the close-range image was that it had only few good features for orientation. Therefore, the maximum error at the direction of the X axis of the ground coordinate system was still almost 7 cm. In addition, some errors in rotations remained. However, when using three images instead of two during the interactive orientation, the overall accuracy of the orientations became better. In the case of the aerial image, the average improvements in maximum errors were 10.3 cm in location and 0.098 degrees in rotations.

In many cases, laser scanning data can be leveled reliably using control patches [52], targets, road markings, linear features or large open planar areas, such as parking areas or football fields. As previous examples have shown, finding accurately rotation differences between images and laser scanning data is a challenging task for interactive orientation. One reason is that the observed area is typically relatively small if terrestrial images are used. In addition, the footprint of the images does not necessarily cover a sufficient number of clear corresponding features to ensure reliable determination of all three rotations. In the last experiment of the interactive orientation, the same three images as in the previous example were used. However, the orientation differences with laser scanning data included only shifts and no rotations. As can be seen from Table 3, the interactive orientation produced results very similar to those of the reference orientation.

After an interactive orientation the errors in rotations are automatically compensated with shifts of the projection center. As a result, even if the image orientations slightly differ from the reference orientation, the effect on the ground is typically much smaller. Correspondingly, the effect on the image plane is typically very small. Therefore, laser scanning data fits locally with the images. In order to detect better small errors in shifts and rotations, a larger area should be examined or more images should be included in the interactive orientation. One solution could be that 2–3 separate image blocks from the different sides of an aerial image are created and registered with laser scanning data.

### 4.2. Integrated Multi-source Data

**Figure 8.**
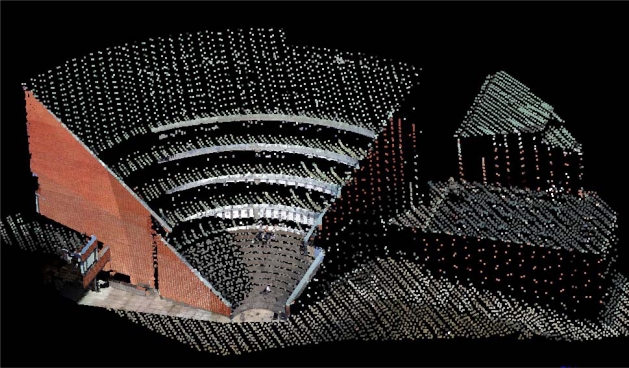
After the orientations, 3D points from photogrammetric measurements, terrestrial laser scanning and airborne laser scanning were integrated. 3D points were colorized using both aerial images and terrestrial panoramic image.

After the orientations, airborne laser scanning data, part of one terrestrial laser scanning data and photogrammetrically-derived 3D points were integrated. In addition, point clouds were colorized using both aerial and terrestrial panoramic images (Figure 8). Terrestrial laser scanning data is easy to differentiate from airborne laser data because of its superior point density.

### 4.3. Confirmation of the Inverse Transformation from Image Orientations to Laser Scanning Point Cloud Transformations

Inverse transformation (Section 3.4) is the final step of the workflow if interactive orientation is applied and laser data is required to be introduced into the original coordinate system of an image block. An experiment was carried out in which an image included calibration targets and the point cloud included target observations from an automatic camera calibration in iWitness software.

**Figure 9.**
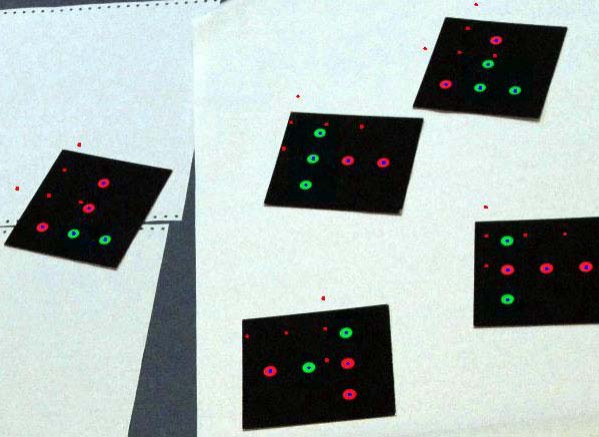
Small red dots illustrate the initial orientation and blue dots the registration after the interactive orientation and the inverse transformation of 3D points according to the relative orientation parameters.

Initially, the test image and the 3D point cloud were at different coordinate systems. Because the interpretation of targets in the image was simple, only a single image was used when an interactive orientation between 3D points and the image was carried out. After the interactive orientation, the image was oriented into the same coordinate system as the 3D points. According to the relative orientation that was solved using the interactive orientation, the 3D points were transformed into the coordinate system of the initial camera pose. Figure 9, on one hand, illustrates the misfit between the 3D points and the targets on images when the initial orientations were used while superimposing the points onto the image (small red dots). On the other hand, the blue dots in the figure show how 3D points are again well registered with the image after the interactive orientation and the inverse transformation.

## 5. Conclusions

Integration of multi-source data requires accurate relative orientations. In this article, an interactive method for registering multi-view, multi-scale image blocks with laser scanning data was presented. During an interactive orientation of an image block, the image orientation parameters of any image from the block can be changed. When an orientation of one image is changed, new orientations are calculated for other images according to the original relative orientation. Laser scanning data is superimposed onto all images using new orientations, which reveals visually the quality of the relative orientation.

Results from the interactive orientation were compared with the orientations of a reference image block, which was calculated using a bundle block adjustment. The laser scanning point cloud, which was fixed during interactive orientation, was pre-registered with a 3D model that was measured photogrammetically using the reference image block. In other words, the reference image block and laser scanning data were in the same coordinate system. Therefore, image orientations after the interactive orientation were comparable with reference orientations.

The first example included an aerial image and a terrestrial panoramic image. The interactive orientation was done eight times, resulting in a maximum shift of 30.5 cm and a maximum rotation difference of 0.194 degrees. In the second example, a terrestrial close-range image was added to the image block to be used for the interactive orientation. As a result, the differences with the reference orientation decreased significantly. At this time, the maximum shift was 20.9 cm and the maximum rotation was 0.105 degrees. The last example included the same images as in the previous experiment. However, the initial image block orientation had no rotation differences compared to the laser data orientation, only shifts. Because the interpretation of laser data became easier when no rotations needed to be solved, the maximum shift was only 2.6 cm.

Typically, images are easier than airborne laser scanning data to orient with regards to the ground coordinate system. Interactive orientation, however, is more flexible if all orientation changes are done to the image orientation parameters. The equations for transferring laser data to the original coordinate system of an image block according to the relative orientation results from the interactive orientation were presented.

Because the accuracy of an interactive orientation is highly dependent on the image block geometry, on the number of images and on the amount of distinguishable features within the image footprints, extensive numerical results cannot be generalized. The area for an interactive orientation should be selected carefully in order to capture many clear features in the images. In areas, in which the amount of distinguishable features is low, thus posing a challenge, the interpretation skills of an operator become significant. The results, however, verify that including more images to interactive orientation increases accuracy. In addition, if laser scanning data is already leveled, an interactive orientation can provide very accurate orientation.

To conclude, interactive orientation improves the quality of multi-source data integration and thus the quality of the final products. However, the cost-effectiveness of the approach in practical applications has to be separately studied. Applications, where, e.g., sparse laser-scanning point clouds are densified with photogrammetrically-generated point clouds, accurate registration of the data can totally dominate the usability of the data, and, thus the highest quality approaches are expected to be needed.

## Figures and Tables

**Table 1.** Differences of exterior orientation parameters (interactive orientation – reference). The interactive orientation was applied using simultaneously a terrestrial panoramic image and an aerial image, whose relative orientation was known. Statistics were calculated from 8 individual orientations.

	**Aerial image**					
	**X (cm)**	**Y (cm)**	**Z (cm)**	*ω***(deg)**	*φ***(deg)**	*κ***(deg)**

Average	−8.1	2.2	−1.1	−0.062	−0.012	0.046
Std	10.6	15.4	2.5	0.064	0.041	0.038
Max	20.0	30.5	5.6	0.194	0.069	0.106
	
	**Panoramic image**					
	**X (cm)**	**Y (cm)**	**Z (cm)**	*ω***(deg)**	*φ***(deg)**	*κ***(deg)**

Average	−3.1	−2.8	−0.6	−0.005	−0.063	0.043
Std	5.1	7.2	1.8	0.055	0.056	0.033
Max	10.2	18.5	4.1	0.101	0.176	0.065

**Table 2.** Differences of exterior orientation parameters (interactive orientation – reference). The interactive orientation was applied using simultaneously a close-range normal-angle image, a terrestrial panoramic image and an aerial image, whose relative orientations were known. Statistics were calculated from 8 individual orientations.

	**Aerial image**					
	**X (cm)**	**Y (cm)**	**Z (cm)**	*ω***(deg)**	*φ***(deg)**	*κ***(deg)**

Average	−9.6	1.5	−0.4	−0.033	−0.017	0.038
Std	7.4	12.0	1.2	0.031	0.036	0.021
Max	20.9	16.3	2.4	0.097	0.065	0.065
	
	**Panoramic image**					
	**X (cm)**	**Y (cm)**	**Z (cm)**	*ω***(deg)**	*φ***(deg)**	*κ***(deg)**

Average	0.6	0.7	−1.2	0.024	−0.017	0.045
Std	2.6	0.4	1.4	0.035	0.022	0.028
Max	6.8	1.3	2.8	0.065	0.046	0.078
	
	**Close-range image**					
	**X (cm)**	**Y (cm)**	**Z (cm)**	*ω***(deg)**	*φ***(deg)**	*κ***(deg)**

Average	1.2	0.3	−0.6	−0.034	0.022	0.008
Std	2.3	0.2	0.8	0.062	0.023	0.050
Max	6.5	0.6	2.0	0.105	0.054	0.097

**Table 3.** Differences of shifts (interactive orientation – reference). Because there were no rotation differences between laser scanning data and the image block coordinate system, the differences of shifts were the same for all images. Statistics were calculated from 8 individual orientations.

	**Image block**		
	**X (cm)**	**Y (cm)**	**Z (cm)**
Average	0.7	0.3	−0.9
Std	1.2	0.3	0.5
Max	2.6	0.6	1.8
